# Adenocarcinoma Arising from Unilateral Retinal Pigment Epithelium Dysgenesis

**DOI:** 10.1177/24741264261467263

**Published:** 2026-07-08

**Authors:** Brendan K. Tao, Andrew Mihalache, Filiberto Altomare, Zelia M. Correa, Eduardo V. Navajas

**Affiliations:** 1 Department of Ophthalmology & Vision Sciences, University of Toronto, Toronto, ON, Canada; 2 Temerty Faculty of Medicine, University of Toronto, Toronto, ON, Canada; 3 Bascom Palmer Eye Institute and Sylvester Comprehensive Cancer Center, University of Miami, FL, USA; 4 Department of Ophthalmology and Visual Sciences, University of British Columbia, Vancouver, BC, Canada

**Keywords:** adenocarcinoma, retinal pigment epithelium, dysgenesis, oncology, pathology

## Abstract

**Purpose:**

To describe the clinical evolution of adenocarcinoma arising from unilateral retinal pigment epithelium (RPE) dysgenesis.

**Methods:**

A single case was reviewed.

**Results:**

A young patient with no relevant family history was referred for evaluation of a fundus lesion of the left eye that had progressed after 5 years of stability. Examination and multimodal imaging revealed a smooth, dome-shaped, amelanotic mass in the posterior pole with overlying vascular distortion and internal calcification. Due to the poor visual prognosis, diagnostic enucleation was performed. Histopathologic analysis revealed a well-differentiated epithelial tumor arising from the RPE. Immunohistochemistry was positive for vimentin and neuron-specific enolase and weakly positive for HMB-45 and MART-1, while electron microscopy demonstrated intercellular basal lamina and desmosome-like junctions, confirming RPE differentiation.

**Conclusions:**

This case illustrates confirmed adenocarcinoma arising from unilateral RPE dysgenesis and supports the role of surveillance for some RPE lesions in which malignant transformation may occur as a rare event.

## Introduction

Primary adenocarcinoma of the retinal pigment epithelium (RPE) is an exceedingly rare intraocular malignancy that may clinically mimic choroidal melanoma, choroidal hemangioma, or melanocytoma.^1^ Most reported cases of RPE adenocarcinoma in adults occur de novo or arise from congenital hypertrophy of the RPE^2,3^; however, biopsy-proven cases developing in the context of unilateral RPE dysgenesis have not previously been reported.^4^

Unilateral RPE dysgenesis is a rare, benign retinal disorder characterized by a flat, dark pigmented lesion with spiculated margins and a distinctive inversion pattern between fundus autofluorescence and fluorescein angiography (FA).^5^ Herein, we describe the long-term clinical outcome, multimodal imaging, and histopathologic and ultrastructural features of RPE adenocarcinoma arising from unilateral RPE dysgenesis in a young patient, highlighting its indolent course and rare malignant transformation.

## Case Report

**Figure 1.**
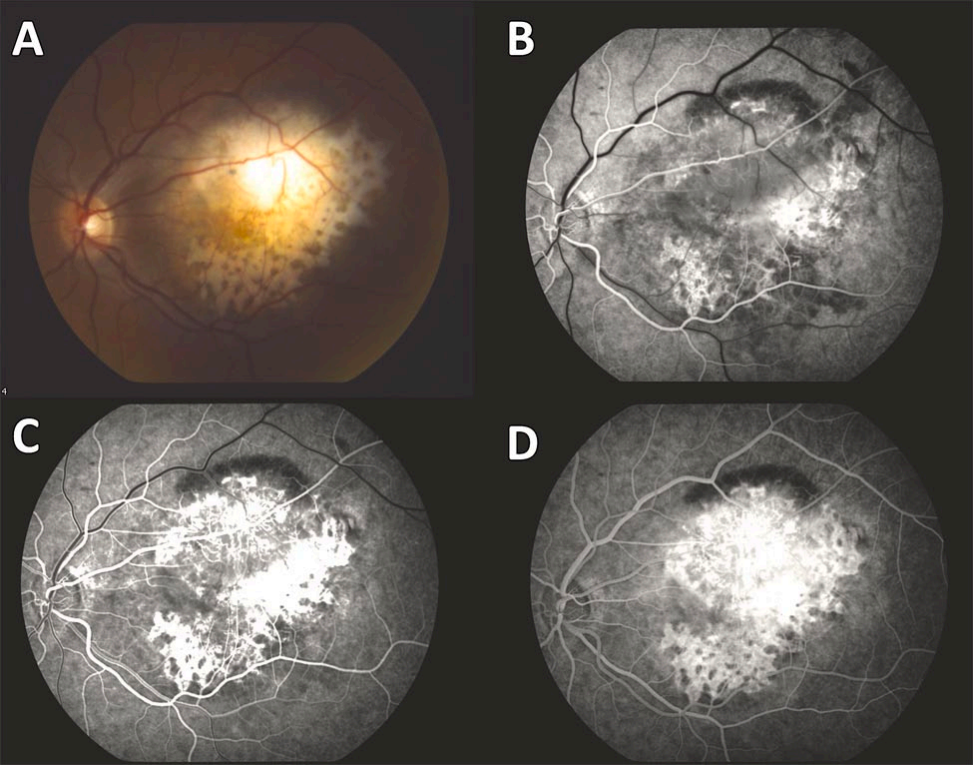
(A) Color fundus photograph of the left eye of a 14-year-old girl at first presentation. The image demonstrates a partially amelanotic lesion with a honeycomb pattern and spiculated borders, predominantly in the temporal and inferior portions. (B–D) Fluorescein angiography of the left eye reveals staining of the inferior and temporal lesion margins and blocking of background fluorescence in the more heavily pigmented areas of the honeycomb pattern and in the superior margin of the lesion. Telangiectatic retinal vessels overlying the lesion are also observed.

A patient in her early twenties was referred for evaluation of an enlarging fundus mass in the left eye. She had no relevant medical or family history. Ocular history was significant for a fundus lesion in the left eye that was first noted at age 14 (Figure 1, A–D). At that time, the lesion was partially amelanotic and demonstrated a honeycomb pattern with spiculated borders, mostly along its temporal and inferior margins (Figure 1A). FA revealed staining of the inferior and lateral lesion margins and blocking of background fluorescence in the more heavily pigmented areas within the honeycomb pattern and along the superior margin of the lesion (Figure 1, B–D). At this stage, telangiectatic retinal vessels overlying the lesion were also evident on FA. The lesion was subsequently monitored by the referring institution with a presumed diagnosis of choroidal osteoma.

The lesion remained stable over 5 years until age 19, when nodular growth was first noticed by the referring institution (Supplemental Figure 1A) and subsequently observed. While FA performed 5 years earlier demonstrated only overlying telangiectatic vessels, repeat FA showed retinal vessels diving into and out of the lesion, consistent with feeding and draining retinal vessels that supplied blood to the lesion.

By age 21, the patient was referred to our tertiary care center due to continued growth in her left eye. On examination, left-sided leukocoria was present, and her best-corrected visual acuity was 20/20 OD and 20/400 OS. Anterior segment examination of both eyes was unremarkable. Serous subretinal fluid (SRF) surrounding the lesion, associated retinal dragging, and vitreous cells were present (Supplemental Figure 2). Fundus examination of the left eye also revealed a hyperpigmented area in the inferonasal periphery, most consistent with vitreoretinal traction (Supplemental Figure 3).

**Figure 2.**
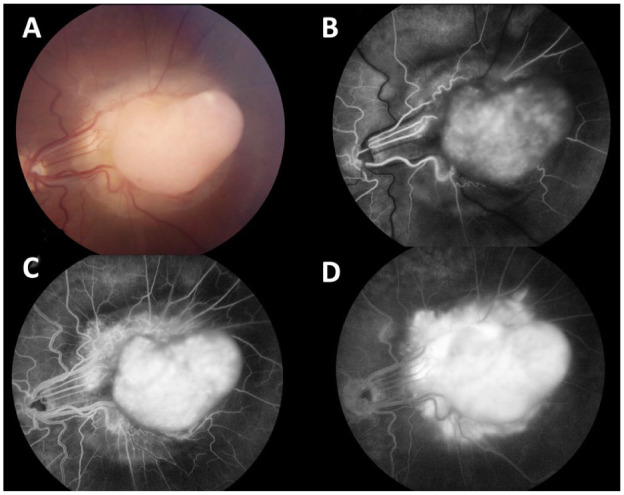
(A) Color fundus photograph of the patient’s left eye obtained at 7-year follow-up. The image demonstrates a large amelanotic lesion with a peripheral component that seems confined to the retina and a central, fleshy, amelanotic nodular eruption protruding temporally from the lesion. (B–D) Intravenous fluorescein angiography during the early, middle, and late phases demonstrates diffuse late leakage, which is more pronounced than that observed 2 years earlier. Feeding and draining retinal vessels associated with the lesion are also evident.

**Figure 3.**
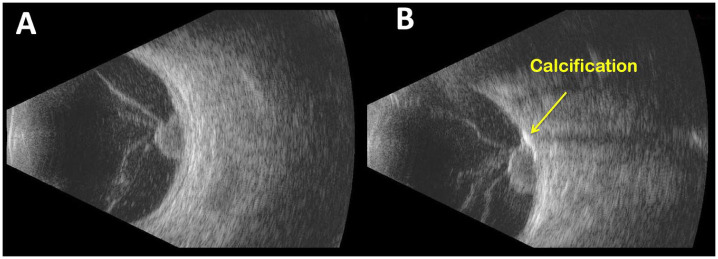
(A) Transverse B-scan ultrasonography of the left eye demonstrating a large, dome-shaped, acoustically solid intraocular mass arising from the posterior pole. The lesion exhibits a characteristic Derby hat configuration. No orbital or extrascleral extension is observed. (B) Magnified view of panel A demonstrating basal calcification associated with posterior acoustic shadow.

**Figure 4.**
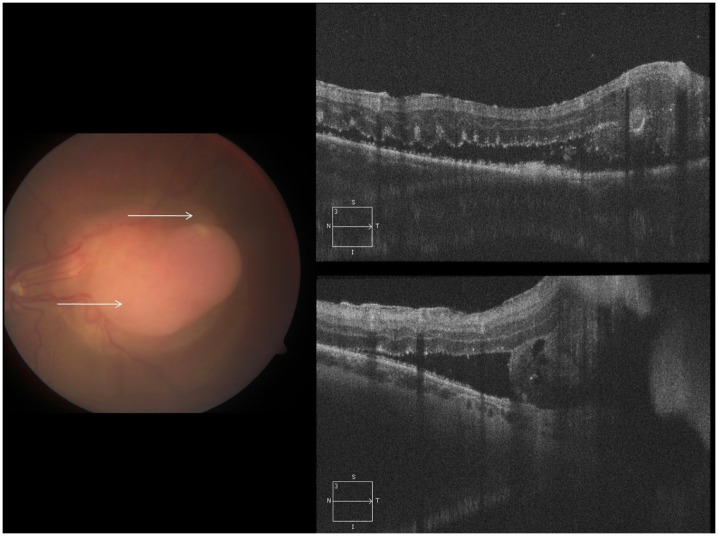
Spectral-domain optical coherence tomography obtained through the superior and inferior margins of the lesion confirmed the presence of subretinal fluid and deposits, outer retinal folds, and a subretinal mass arising from the retinal pigment epithelium.

More notably, fundus examination revealed a large amelanotic lesion with a peripheral component that seemed confined to the retina and a central amelanotic, fleshy nodular eruption protruding temporally from the lesion (Figure 2A). Similar findings had been noted 2 years earlier (Supplemental Figure 1A) but were absent when the lesion was first identified (Figure 1A). FA again demonstrated diffuse late leakage with feeding and draining lesional vessels (Figure 2, B–D), findings that were more pronounced than those observed 2 years earlier (Supplemental Figure 1, B–D). B-scan ultrasonography of the left eye demonstrated a solid, Derby hat-shaped tumor measuring 3.5 mm in thickness, with basal calcification generating an acoustic shadow (Figure 3, A–C). Spectral-domain optical coherence tomography obtained through the superior and inferior margins of the lesion confirmed the presence of SRF and deposits, outer retinal folds, and a mass in the subretinal space contiguous with the retina that did not appear to involve the choroid (Figure 4 and Supplemental Figure 4). The underlying choroid was unremarkable. A summary of the longitudinal ocular imaging findings is provided in Supplemental Figure 5.

Given the poor visual prognosis potential and the presence of calcification within a creamy-white retinal lesion showing documented growth, the working diagnosis was malignant transformation of a retinocytoma. After discussion of the available treatment options, the patient elected to undergo enucleation.

**Figure 5.**
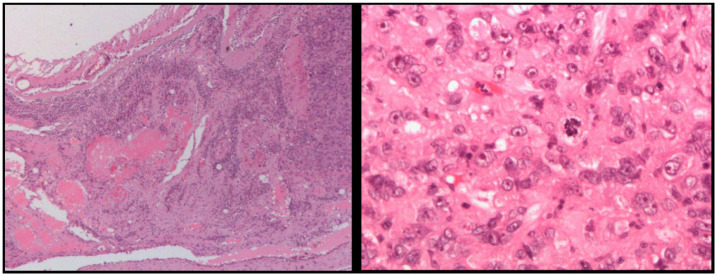
Hematoxylin and eosin-stained sections demonstrating the histopathologic features of the tumor at low- and high-power magnification. The neoplasm is composed of amelanotic cells containing large, hyperchromatic, and pleomorphic nuclei with prominent nucleoli and eosinophilic cytoplasm. Mitotic activity, including atypical mitoses, is present. The tumor cells are seen widely infiltrating the retina and involving the choroid, with focal tubular differentiation. Reactive retinal pigment epithelium proliferation as well as bone metaplasia are also present. No extraocular extension is identified.

Macroscopically, the sectioned globe showed a dome-shaped, white, abruptly elevated mass measuring 7 mm × 5.5 mm in diameter and 4 mm in thickness (Supplemental Figure 6). Histopathologic examination demonstrated a tumor composed of amelanotic cells with large, hyperchromatic, pleomorphic nuclei, prominent nucleoli, and eosinophilic cytoplasm (Figure 5). Most tumor cells were individually surrounded by membrane-like material that stained positive with periodic acid–Schiff and was resistant to diastase treatment, with some cells having vacuoles within the cytoplasm. Mitotic activity, including atypical mitoses, was detected.

The tumor cells extensively infiltrated the retina and focally involved the choroid. Within the choroid, some cohesive tumor cells formed tubular structures. Reactive RPE proliferation as well as bone metaplasia were observed in some areas. Rare extracellular pigment deposition was noted within the tumor. No extraocular extension was identified. Histopathologic examination further demonstrated continuity between the RPE and the tumor, supporting an RPE origin (Supplemental Figure 7).

A panel of immunohistochemical stains was performed (Supplemental Figures 8–10). The tumor cells were strongly positive for vimentin, neuron-specific enolase, and CD68. The Ki-67 proliferation marker was positive in 5% of the cells. Focal positivity was observed for HMB-45, synaptophysin, cytokeratin 8/18, and alpha-smooth muscle actin. Staining for S100, MART-1, cytokeratin AE1/AE3, epithelial membrane antigen, and glial fibrillary acidic protein was negative.

Transmission electron microscopy revealed no definitive melanosomes or premelanosomes (Supplemental Figure 11). A dense extracellular matrix with a fibrillary component was noted between tumor cells, and similar material was also observed within the cytoplasm of some cells. Ultrastructural features of RPE differentiation were also observed, including intercellular basal lamina deposition and desmosome-like junctions.

The final diagnosis was adenocarcinoma of the RPE, based on the morphologic features, immunohistochemical profile, and electron microscopy findings. In retrospect, the lesion first documented at age 14 (Figure 1, A–D) was considered consistent with unilateral RPE dysgenesis, which subsequently gave rise to a nodular RPE adenocarcinoma.

## Conclusions

Primary adenocarcinoma of the RPE is an exceedingly rare intraocular neoplasm for which enucleation remains the standard of care, albeit local resection may be feasible in select cases.^3,6^ The present case delineates the clinical and pathological evolution of a primary RPE adenocarcinoma arising from a long-standing lesion consistent with unilateral RPE dysgenesis, a benign and nonproliferative retinal disorder not previously implicated in neoplastic transformation.^4^ Over a 7-year follow-up period, serial multimodal imaging demonstrated gradual enlargement and progressive elevation of the lesion, culminating in diagnostic enucleation and histopathologic confirmation of adenocarcinoma. To our knowledge, this is the first reported case demonstrating the histopathologic evolution of an adenocarcinoma arising from unilateral RPE dysgenesis, corroborated by long-term clinical follow-up and comprehensive multimodal imaging. A limitation of this case report is the absence of other imaging modalities, such as optical coherence tomography angiography and fundus autofluorescence, which could have provided additional insights into lesion progression and vascular characteristics.

Throughout the follow-up period, the lesion preferentially invaded and grew inward toward the retina. We propose 3 possible explanations for this growth pattern. First, Bruch membrane, with its dense acellular matrix, may act as a physical barrier to tumor extension, whereas the retina and vitreous represent a path of lesser resistance. Second, Bruch membrane may act as a biochemical barrier, as it contains several antiangiogenic and anti-invasive factors, including endostatin, pigment epithelium-derived factor, and thrombospondin-1, which may support a biochemical milieu that is relatively resistant to tumor invasion and neovascularization.^7^ Finally, as the lesion arises from the RPE, it characteristically recruits retinal arterial blood supply rather than choroidal supply. This vascular dependence may favor growth toward the retina and its associated blood supply.

On November 3, 2025, a search of Ovid MEDLINE and Embase (Supplemental Figure 12) identified 7 relevant articles corresponding to 4 unique case reports. Among these, only 1 report described a presumed malignant transformation of unilateral RPE dysgenesis in a 30-year-old woman.^8^ In that report, Gal-Or et al documented a well-circumscribed subretinal mass arising from a lesion of unilateral RPE dysgenesis. The mass was hyporeflective on optical coherence tomography and associated with SRF.^8^ Over a 5-month follow-up period, intravitreal antivascular endothelial growth factor therapy led to resolution of the subretinal exudation and a modest reduction in tumor thickness. However, unlike the present case, no biopsy or histopathologic confirmation was obtained.^8^

Our literature search did not identify any previously reported case of histopathologically proven adenocarcinoma arising from unilateral RPE dysgenesis.

Other complications previously reported in association with unilateral RPE dysgenesis include retinal folds, epiretinal membrane, macular atrophy, and secondary choroidal neovascularization.^9^ For instance, Preziosa et al^9^ described a 51-year-old woman with unilateral RPE dysgenesis complicated by choroidal neovascularization who was effectively managed with intravitreal bevacizumab, resulting in an improvement of best-corrected visual acuity from 20/200 OS to 20/50 OS after 2 injections. This complication was not observed in our patient, who instead demonstrated malignant transformation at a considerably younger age. Further research is warranted to identify patient- and lesion-specific factors that may predispose individuals with unilateral RPE dysgenesis to vision-threatening complications, including malignant transformation.

Overall, this report broadens the recognized clinical spectrum of unilateral RPE dysgenesis, suggesting, in rare instances, its potential for malignant transformation into primary adenocarcinoma of the RPE. Progressive lesion enlargement, evolving structural changes, or associated visual decline in patients with unilateral RPE dysgenesis should raise clinical suspicion for possible neoplastic transformation and prompt comprehensive ophthalmic evaluation.

An additional challenge posed by this rare entity is the absence of standardized recommendations for metastatic surveillance. Although the metastatic potential of primary adenocarcinoma arising from unilateral RPE dysgenesis appears to be very low, it remains poorly defined.^3^ Consequently, decisions regarding systemic staging and longitudinal surveillance should be individualized and made in conjunction with ocular oncology and medical oncology specialists. Future research focusing on regular long-term surveillance of such lesions is essential to elucidate their natural history and to identify clinical or imaging features that may predict malignant transformation.

## Supplemental Material

sj-pptx-1-vrd-10.1177_24741264261467263 – Supplemental material for Adenocarcinoma Arising from Unilateral Retinal Pigment Epithelium Dysgenesis Supplemental material, sj-pptx-1-vrd-10.1177_24741264261467263 for Adenocarcinoma Arising from Unilateral Retinal Pigment Epithelium Dysgenesis by Brendan K. Tao, Andrew Mihalache, Filiberto Altomare, Zelia M. Correa and Eduardo V. Navajas in Journal of VitreoRetinal Diseases

## References

[1] Williams BK Di Nicola M Acaba-Berrocal LA , et al. Adenoma and adenocarcinoma of the retinal pigment epithelium: a review of 51 consecutive patients. Ophthalmol Retina. 2020;4(8):829-839. doi:10.1016/j.oret.2020.03.008 32417354

[2] Shields JA Shields CL Eagle RC Jr Singh AD. Adenocarcinoma arising from congenital hypertrophy of retinal pigment epithelium. Arch Ophthalmol. 2001;119(4):597-602. doi:10.1001/archopht.119.4.597 11296028

[3] Sreenivasan J Rishi P Das K Krishnakumar S Biswas J. Retinal pigment epithelium adenoma and adenocarcinoma: a review. Ocul Oncol Pathol. 2020;7(2):121-132. doi:10.1159/000509484 33981695 PMC8077658

[4] Cohen SY Lathiere T Marechal V Rodriguez F Souied E Shields CL. Unilateral retinal pigment epithelium dysgenesis (URPED): new cases, literature review, and considerations of the similarities and differences with combined hamartoma of the retina and retinal pigment epithelium (CHRRPE). Retin Cases Brief Rep. 2026;20(4):499-509. doi:10.1097/ICB.0000000000001785 40690768

[5] Cohen SY Fung AE Tadayoni R , et al. Unilateral retinal pigment epithelium dysgenesis. Am J Ophthalmol. 2009;148(6):914-919.e2. doi:10.1016/j.ajo.2009.06.033 19733831

[6] Trichopoulos N Augsburger JJ Schneider S. Adenocarcinoma arising from congenital hypertrophy of the retinal pigment epithelium. Graefes Arch Clin Exp Ophthalmol. 2006;244(1):125-128. doi:10.1007/s00417-005-0011-x 15983818

[7] Bhutto IA Uno K Merges C Zhang L McLeod DS Lutty GA. Reduction of endogenous angiogenesis inhibitors in bruch’s membrane of the submacular region in eyes with age-related macular degeneration. Arch Ophthalmol. 2008;126(5):670-678. doi:10.1001/archopht.126.5.670 18474778 PMC4943079

[8] Gal-Or O Finger PT Fisher YL Yannuzzi LA Freund KB. Presumed retinal pigment epithelium tumor originating from unilateral retinal pigment epithelium dysgenesis. Retin Cases Brief Rep. 2019;13(2):121-126. doi:10.1097/ICB.0000000000000568 28333854

[9] Preziosa C Staurenghi G Pellegrini M. Optical coherence tomography angiography findings in a case of choroidal neovascularization secondary to unilateral retinal pigment epithelium dysgenesis treated with intravitreal bevacizumab therapy. Retin Cases Brief Rep. 2021;15(5):598-601. doi:10.1097/ICB.0000000000000859 30688847

