# Supplementary material for: Adenocarcinoma Arising from Unilateral Retinal Pigment Epithelium Dysgenesis
Source: J Vitreoretin Dis. 2026 Jul 8:24741264261467263. Online ahead of print. doi: 10.1177/24741264261467263 (PMC13346098; doi:10.1177/24741264261467263)
Supplement: sj-pptx-1-vrd-10.1177_24741264261467263 – Supplemental material for Adenocarcinoma Arising from Unilateral Retinal Pigment Epithelium Dysgenesis [file sj-pptx-1-vrd-10.1177_24741264261467263.pptx]

## Slide 1
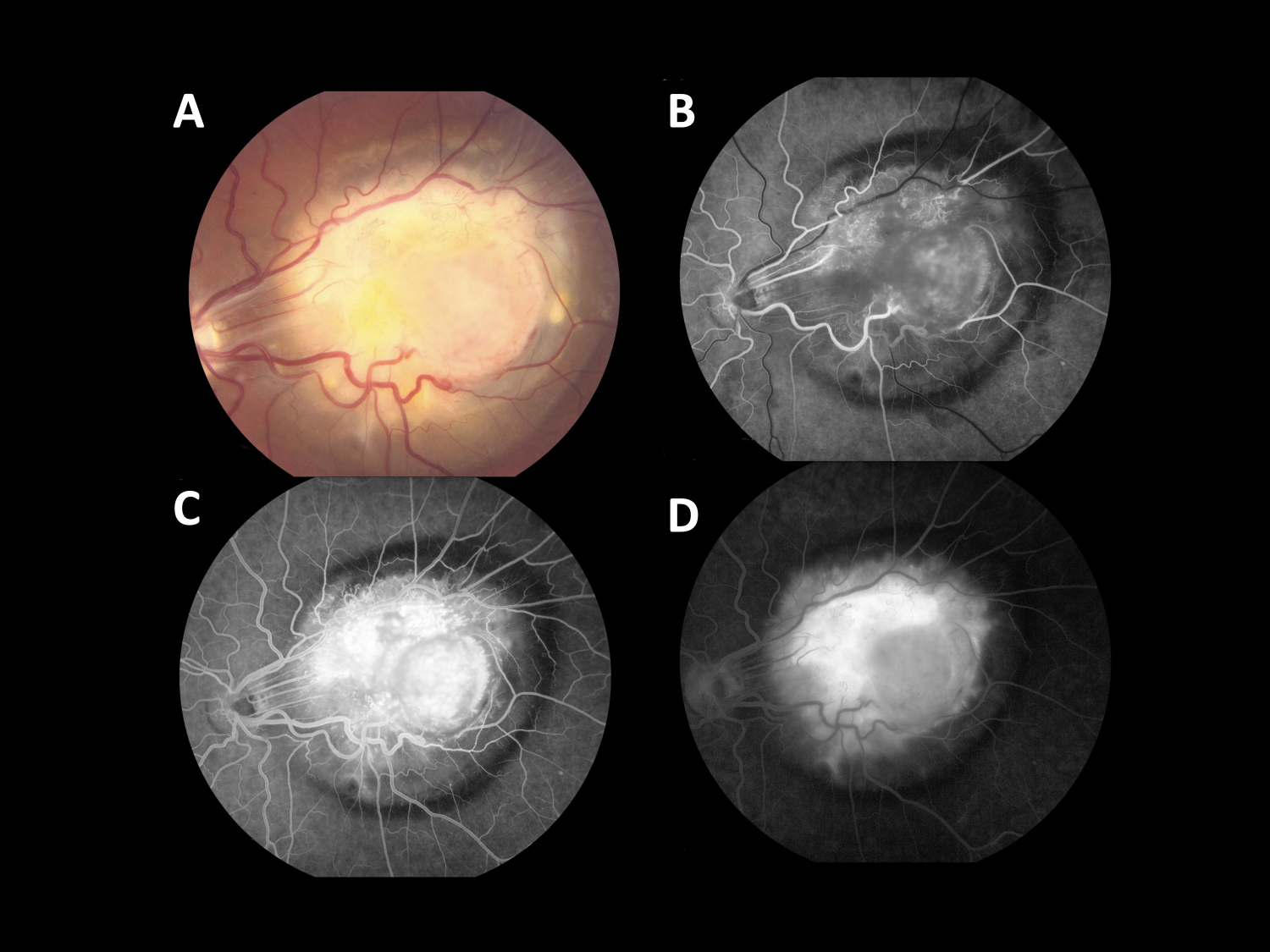

## Slide 2
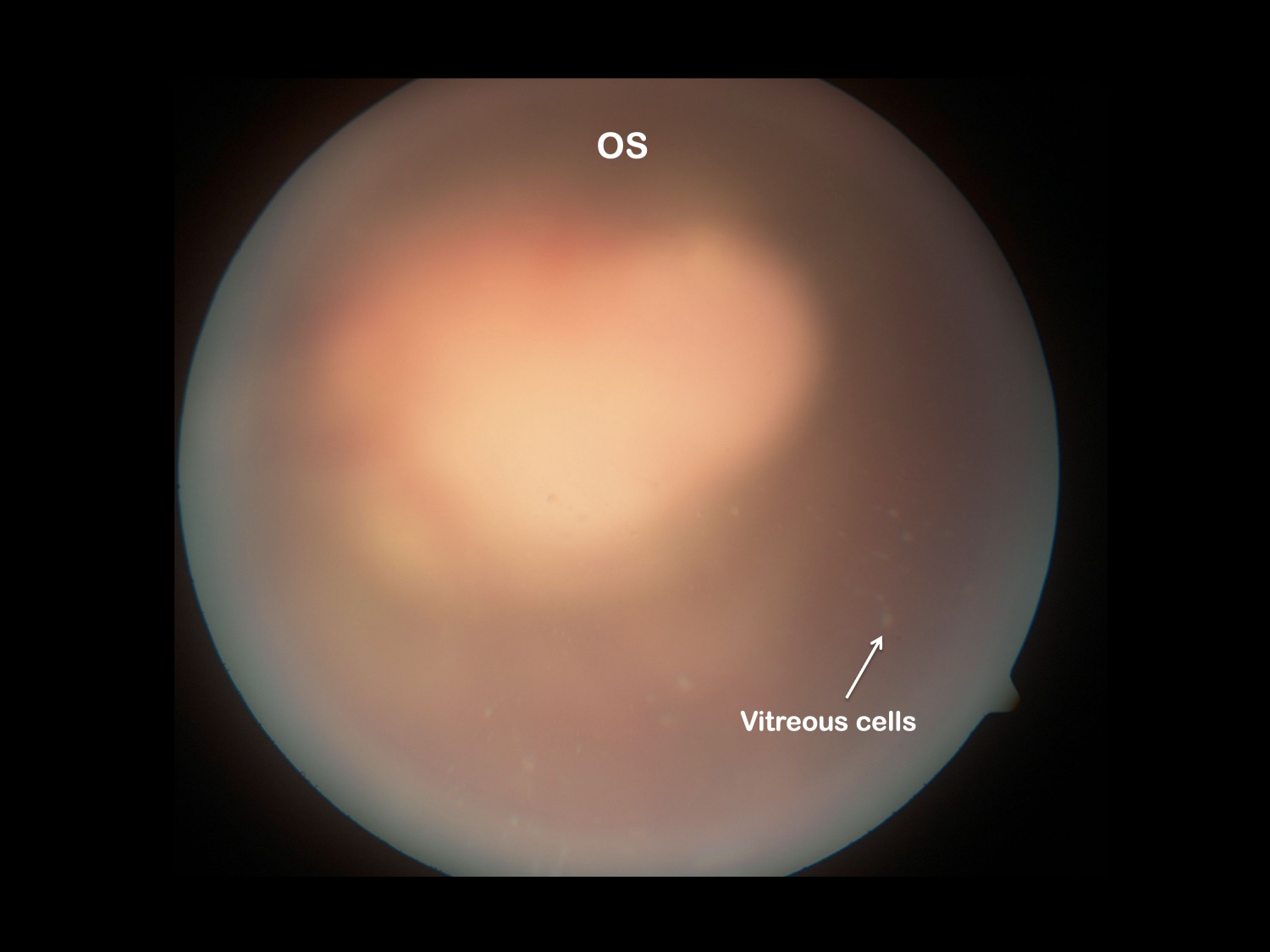

## Slide 3
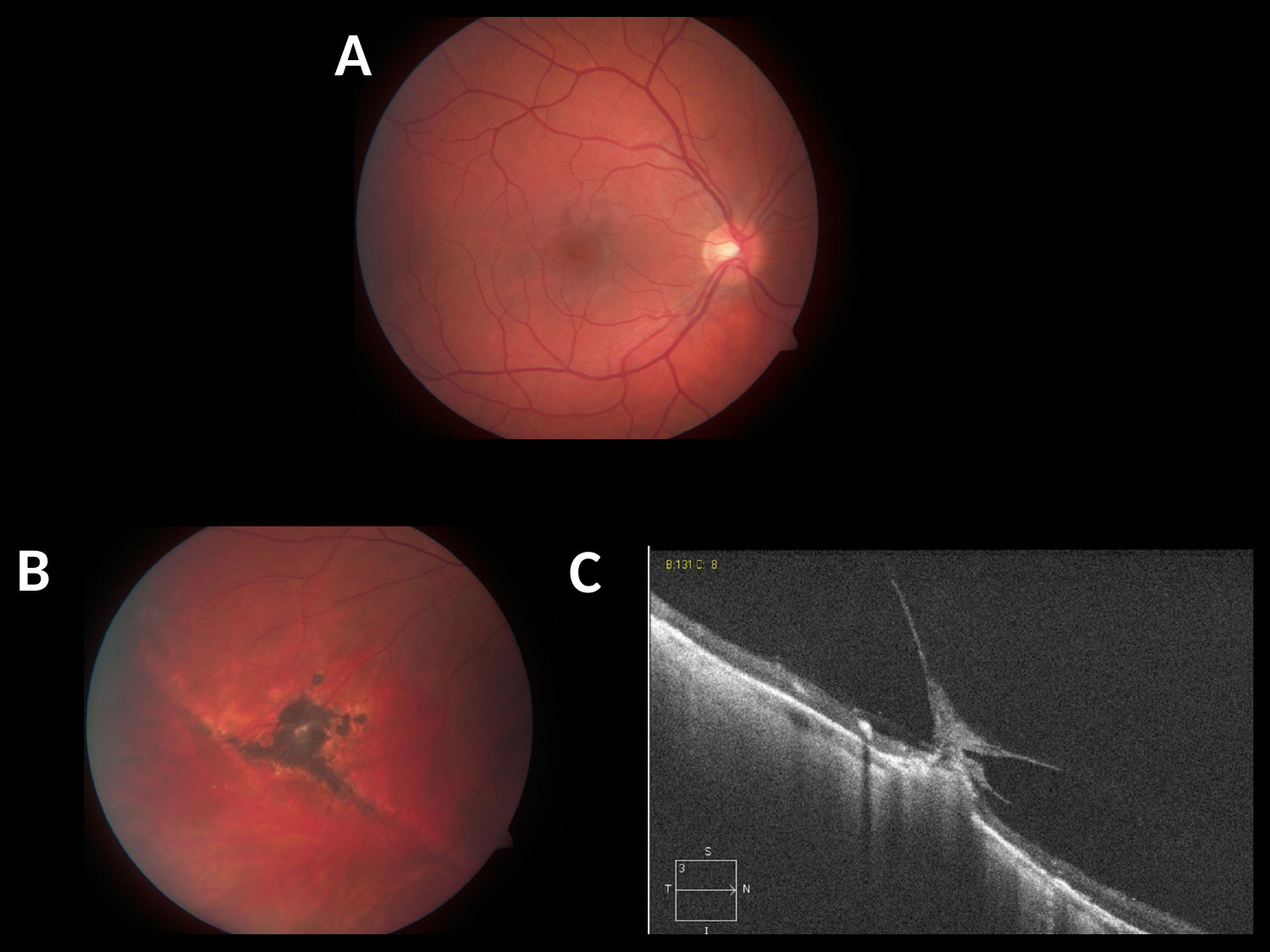

A
B
C

## Slide 4
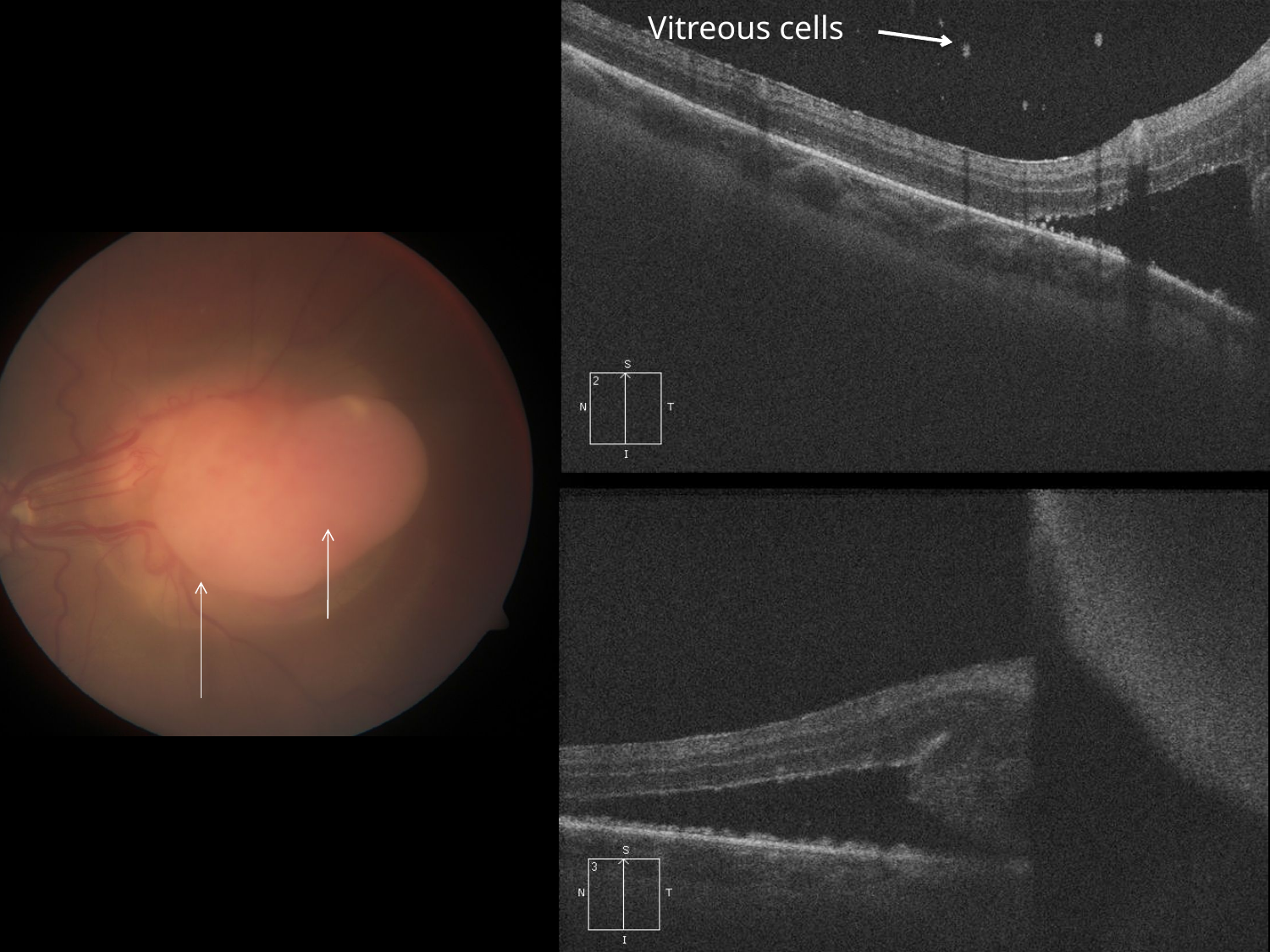

Vitreous cells

## Slide 5
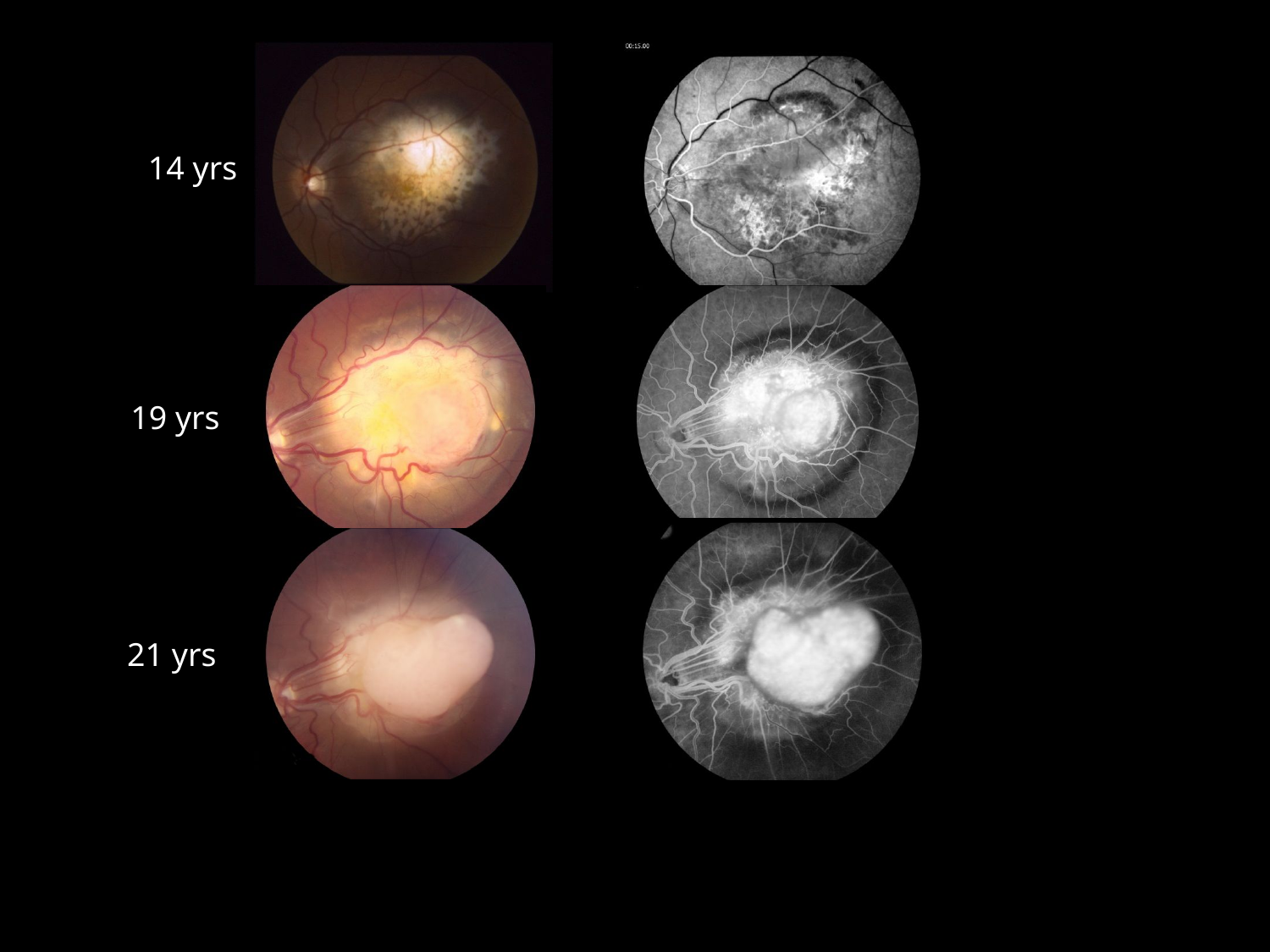

14 yrs
19 yrs
21 yrs

## Slide 6
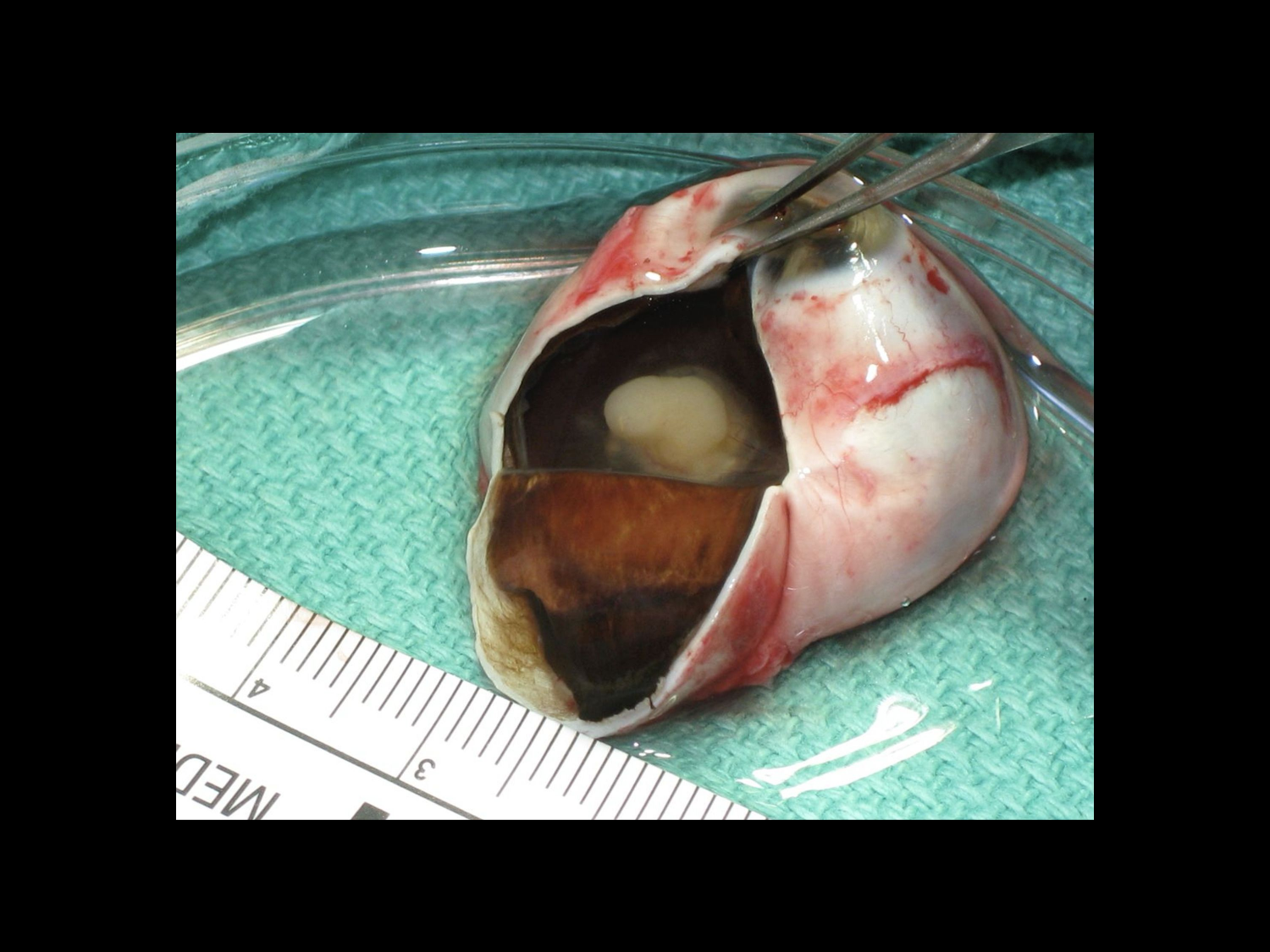

## Slide 7
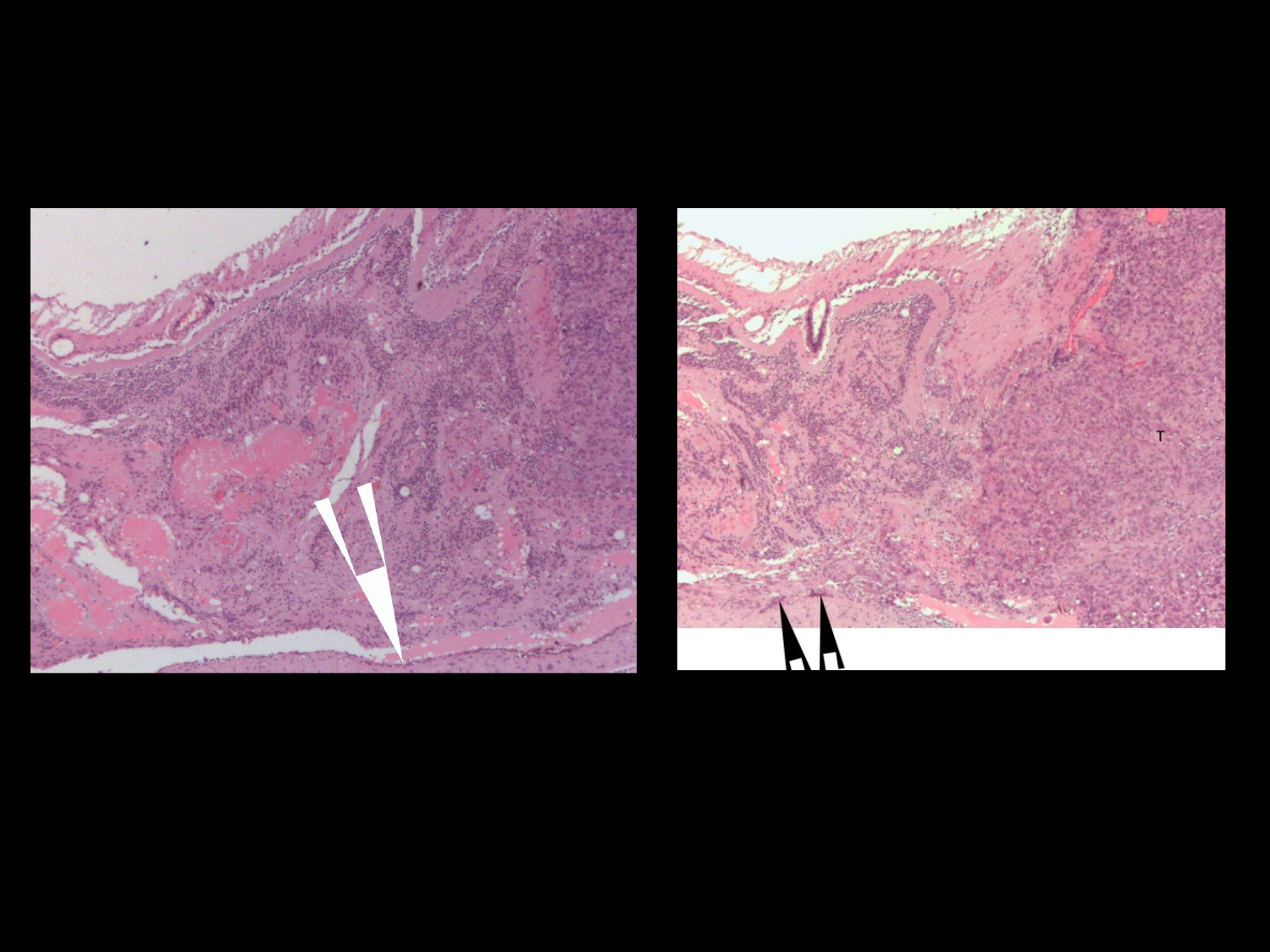

## Slide 8
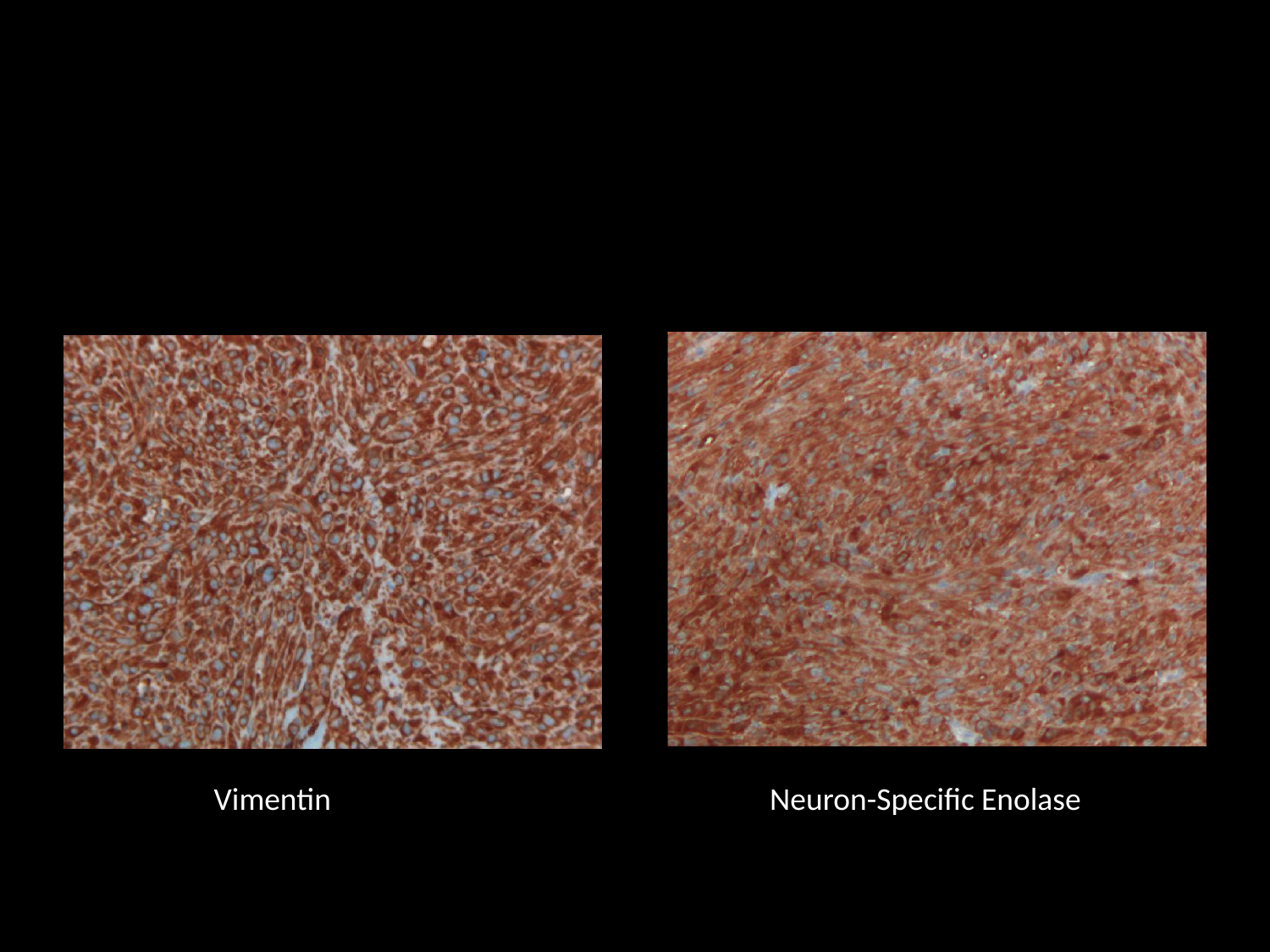

#
Vimentin
Neuron-Specific Enolase

## Slide 9
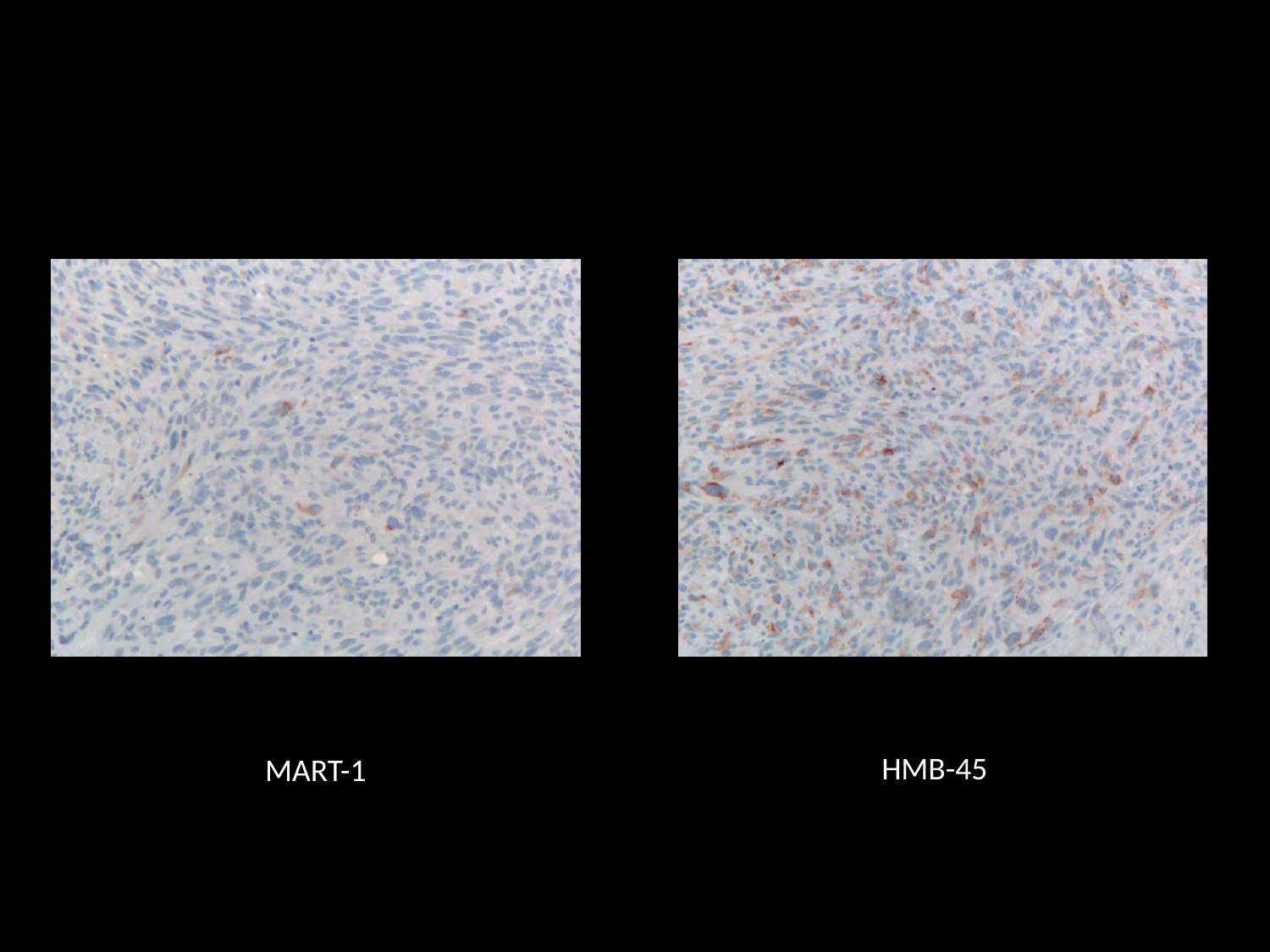

HMB-45
MART-1

## Slide 10
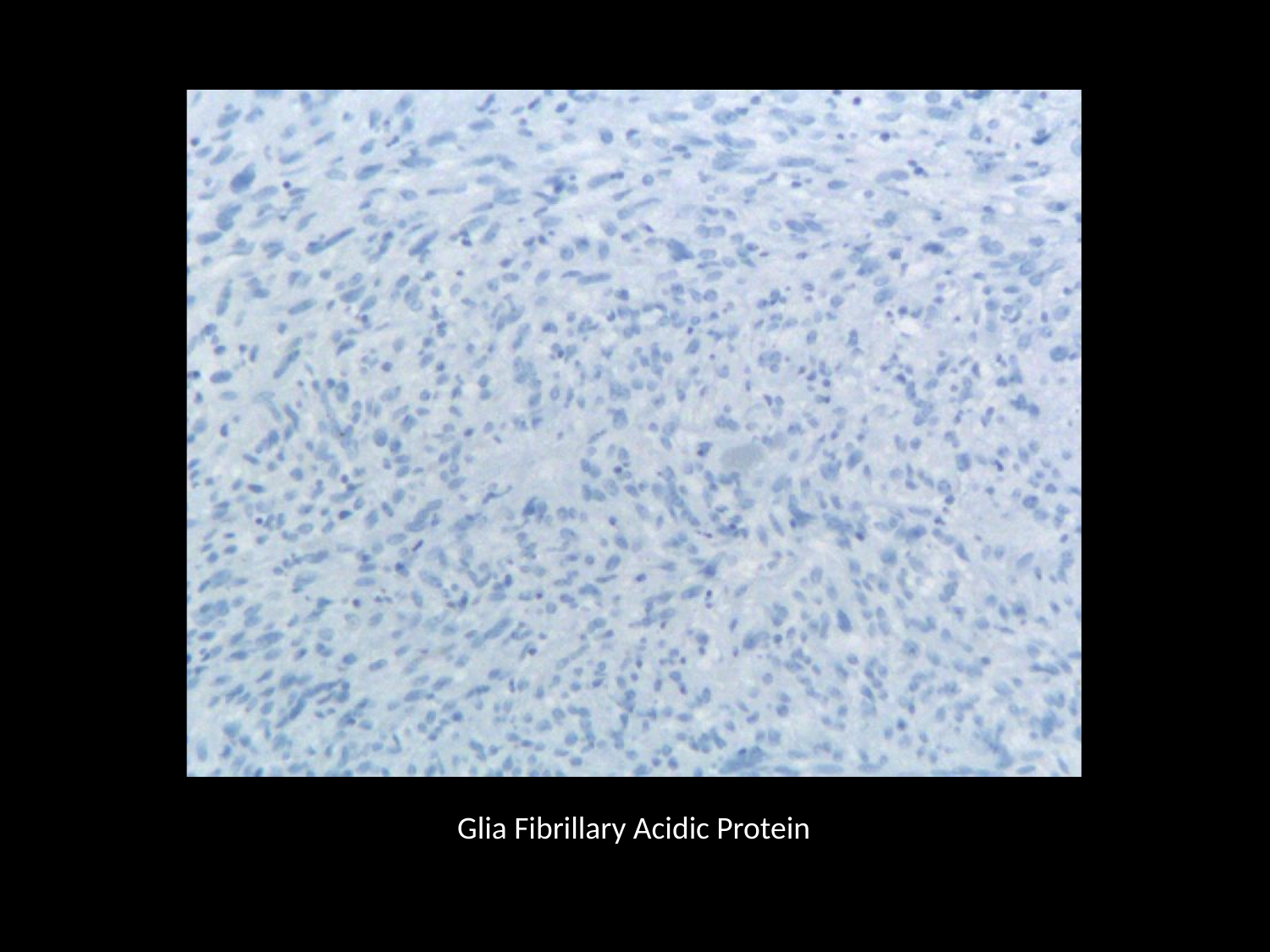

Glia Fibrillary Acidic Protein

## Slide 11
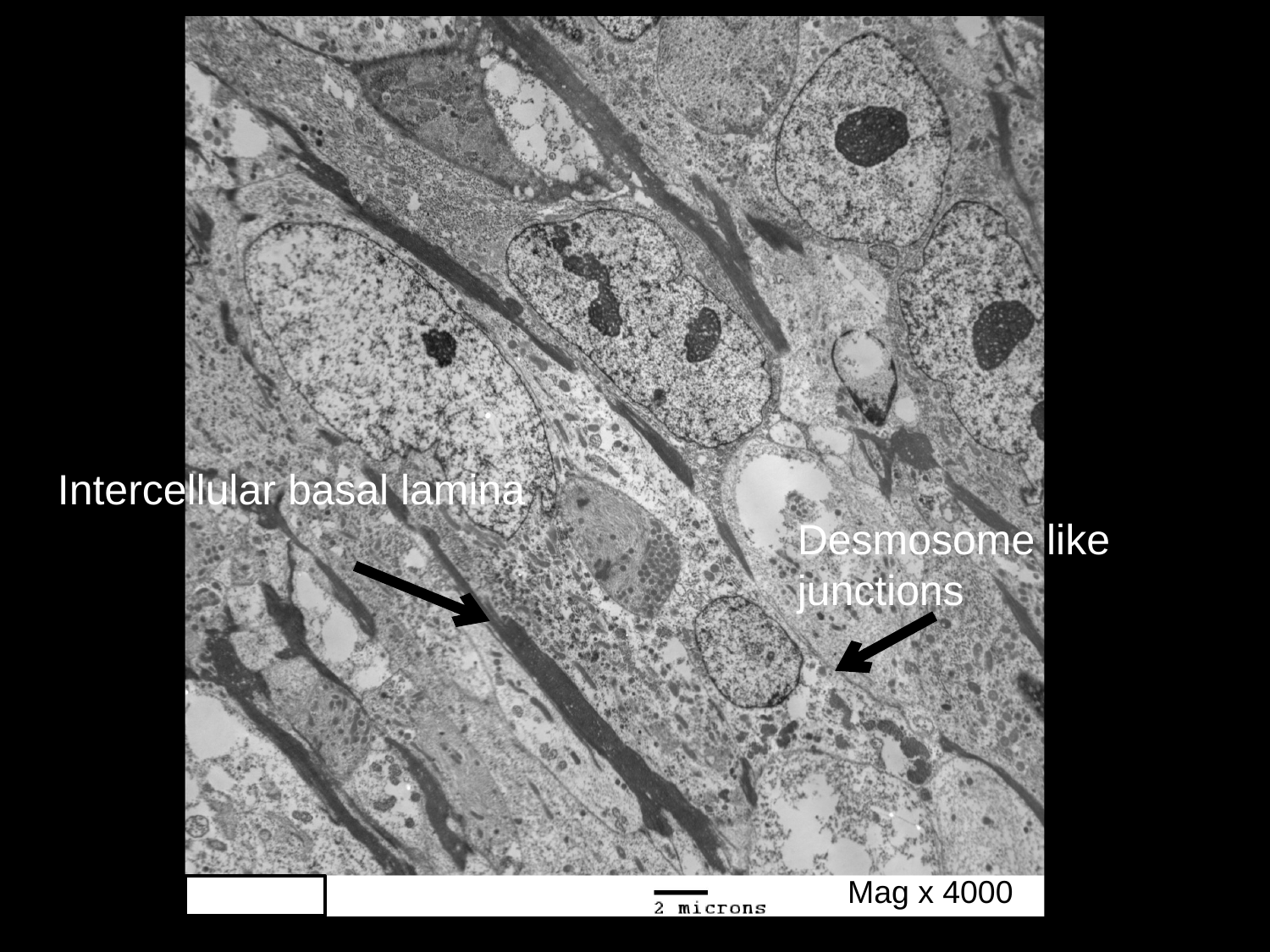

Intercellular basal lamina
Desmosome like junctions
Mag x 4000

## Slide 12
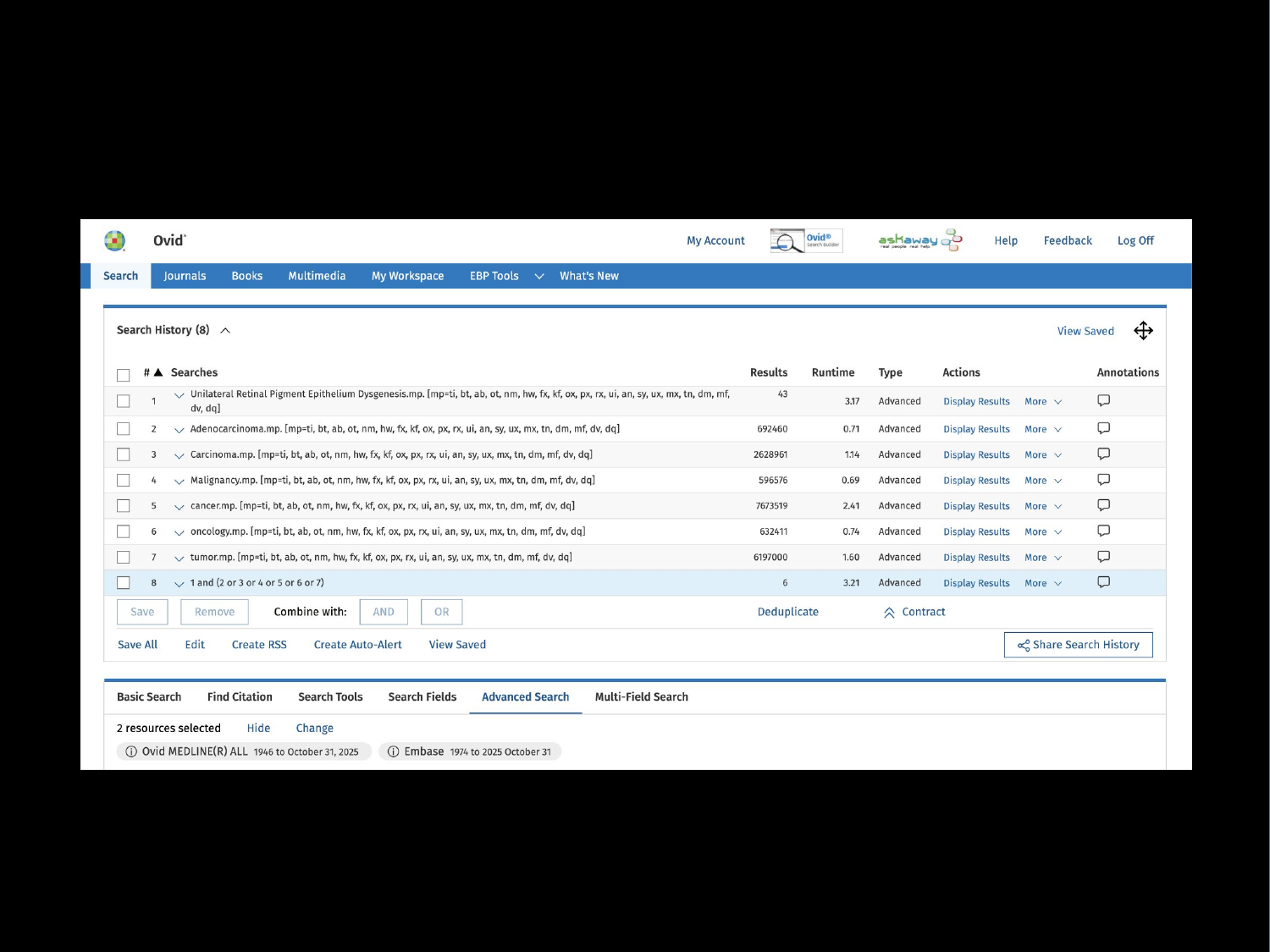

Mag x 4000
